# Behavioural correlates of higher 12-month myopia progression in a high-risk vietnamese schoolchild cohort and the limits of cross-school prediction

**DOI:** 10.1371/journal.pone.0358487

**Published:** 2026-10-06

**Authors:** Sa Huynh Nguyen, Duy Phong Nguyen, Tat Thang Tran

**Affiliations:** 1 Department of Cornea, Nghe An Eye Hospital, Vinh Phu Ward, Nghe An Province, Vietnam; 2 Faculty of Public Health, University of Medicine and Pharmacy at Ho Chi Minh City, Ho Chi Minh City, Vietnam; University of Warmia, POLAND

## Abstract

**Background:**

The clinical interpretation of myopia progression depends on the measurement interval, refractive protocol, and the population from which the analytic sample was selected.

**Methods:**

We performed a secondary analysis of 200 myopic schoolchildren from 10 schools who had been selected for the intervention stage of a parent study after rapid progression (>=1.0 D/year). Parent-study documentation indicates a 12-month follow-up and progression calculated from the change in spherical-equivalent refraction (SE). The de-identified analytic archive retained only three 12-month progression values (1.25, 1.50, and 1.75 D); it did not retain paired eye-level refractions or sufficient metadata to independently verify the exact discretization mechanism or cycloplegic status of both examinations. We therefore contrasted a study-specific higher progression category (>=1.50 D; 99/200, 49.5%) with 1.25 D. A parsimonious logistic model used school-clustered robust standard errors (n = 199 complete cases; 99 outcomes; 10 predictor coefficients), and prediction was evaluated by leave-one-school-out cross-validation.

**Results:**

None of the four questionnaire-derived behavioural indices was independently associated with the higher progression category with stable precision: device-time aOR 1.074 (95% CI 0.993–1.161), outdoor aOR 1.015 (0.929–1.109), near-work aOR 0.972 (0.825–1.145), and reading-risk aOR 1.091 (0.799–1.490). Grade level showed an association (aOR 1.210 per grade, 95% CI 1.099–1.332). Cross-school prediction was poor (AUC 0.540 for the core model and 0.570 after behavioural augmentation; Brier scores 0.252 and 0.254, respectively).

**Conclusions:**

In this selected high-risk cohort, questionnaire-derived behavioural scores did not provide robust adjusted associations or clinically useful cross-school prediction. The findings should not be used for individualized risk stratification or causal behavioural claims. Future studies require prospectively documented cycloplegic refraction, axial length, treatment data, objective exposure measures, and a larger number of schools.

## 1 Introduction

Myopia has emerged as one of the most important paediatric eye-health challenges worldwide because onset in childhood is commonly followed by years of progression, increasing the lifetime probability of high myopia and its vision-threatening sequelae. Contemporary syntheses show that childhood and adolescent myopia prevalence has risen markedly over recent decades and is projected to continue increasing through mid-century, underscoring a growing service and prevention burden for families, schools, and health systems [1]. At the population level, forecasts of overall myopia and high myopia highlight the scale of the problem and the downstream implications for myopia-related ocular complications and avoidable visual impairment [2]. Importantly, the clinical significance of myopia is not limited to refractive correction: high myopia is strongly linked to pathological changes of the posterior segment (e.g., myopic maculopathy), which can cause irreversible vision loss; the International Myopia Institute (IMI) has emphasized the need to address progressive myopia early to mitigate later-life disease risk [3].

Alongside genetic susceptibility, modifiable behavioural and environmental factors play a central role in myopia development and progression. The IMI risk-factor report identifies education-related visual demands and time outdoors as the most consistently supported causal exposures, while noting ongoing challenges in measuring near-work and screen behaviours reliably in real-world settings [4]. A large body of evidence links higher near-work exposure with greater odds of myopia, with meta-analytic estimates indicating a small but consistent association and suggesting cumulative near visual load as a meaningful lever for prevention strategies [5]. In contrast, time outdoors appears protective: systematic reviews report lower risk of prevalent and incident myopia with greater outdoor exposure [6], and school-based randomized evidence demonstrates that increasing time outdoors during the school day can reduce the incidence of myopia in children [7]. In recent years, rapid digitalization has intensified attention on screen-related near work. Earlier evidence syntheses suggested that smart device exposure may be associated with myopia but were limited by heterogeneity and measurement bias in screen-time assessment [8]. More recently, an updated dose–response meta-analysis reported a monotonic increase in myopia odds with increasing digital screen time and suggested a non-linear risk pattern across common exposure ranges, renewing the public-health relevance of screen exposure as a potentially modifiable behaviour among children and adolescents [9]. Together, these findings motivate context-specific, school-anchored evidence that can quantify how near-work, screen use, and outdoor time jointly relate to myopia outcomes—especially in settings where educational intensity and device access are changing quickly.

Vietnam is an illustrative example where school-aged myopia is increasingly recognized as a pressing public-health concern, yet locally actionable evidence remains limited. A recent meta-analysis of Vietnamese schoolchildren synthesized available prevalence data and highlighted a substantial refractive-error burden in the classroom setting, reinforcing the need for prevention and early management strategies tailored to Vietnam’s educational and social context [10]. However, much of the available literature in Vietnam and comparable settings remains focused on descriptive prevalence or single-level association analyses. Two methodological gaps are particularly important for translating evidence into school-based action. First, school environments shape daily schedules, teaching intensity, opportunities for outdoor activity, and informal norms around device use; therefore, students are naturally clustered within schools, and risk-factor associations may vary by school context. Ignoring this multilevel structure can lead to biased inference (e.g., underestimated uncertainty) and can obscure higher-level leverage points for intervention. Second, there is increasing demand not only for etiologic inference but also for practical prediction: identifying students at higher risk of faster progression could support targeted counselling, school-level scheduling modifications, and referral pathways. Yet many prediction efforts in behavioural epidemiology rely on random train–test splits that unintentionally leak cluster-level information across folds, producing overly optimistic performance estimates and reducing transportability when models are applied to new schools.

To address these gaps while respecting the constraints of the available archive, this study had two complementary aims. First, we estimated parsimonious, cluster-aware associations between questionnaire-derived behavioural indices and a higher recorded 12-month progression category within a selected high-risk subgroup. Second, we evaluated whether the available demographic, school-context, and behavioural variables could discriminate that higher category when transported to an unseen school. The prediction analysis was treated as a validation question rather than as development of an implementable clinical tool, and reporting was guided by STROBE and TRIPOD principles [11,12].

## 2 Materials and methods

### 2.1 Study design, parent-study context, and setting

This was a secondary analysis of a de-identified analytic dataset containing 200 myopic schoolchildren clustered within 10 schools and five grade levels (S1 File). Parent-study documentation describes a longitudinal school-based program in Nghe An Province in which myopic pupils were re-examined after 12 months using the same vision and refraction workflow as at baseline. Progression was calculated from the change in spherical-equivalent refraction (SE) over that 12-month interval. For the subsequent intervention stage, 200 pupils were purposively selected from those meeting the parent-study rapid-progression criterion (>=1.0 D/year). Consequently, the present dataset represents a range-restricted, high-risk subgroup rather than an unselected sample of all myopic schoolchildren. This secondary analysis was reported in accordance with STROBE, with the prediction component additionally mapped to TRIPOD reporting principles [11,12].

### 2.2 Participants and analytic eligibility

All 200 records in the supplied optimized dataset had a recorded progression outcome and school identifier. The principal association analysis used complete cases for the prespecified covariates; one participant (0.5%) lacked parental-myopia information, yielding n = 199. Outcomes were not imputed. Because the intervention-stage archive does not contain the original screening denominator or a complete reconstruction of the selection process beyond the parent-study criterion described above, the present analysis does not estimate population prevalence or incidence.

### 2.3 Outcome definition and measurement provenance

Parent-study documentation indicates that myopia progression was assessed over 12 months and calculated as the difference in SE between baseline and follow-up. In the supplied de-identified analytic archive, however, myopia_progression contains only three retained values: 1.25 D (101 students), 1.50 D (94), and 1.75 D (5). Paired eye-level sphere/cylinder or continuous SE measurements, examination dates, an eye-selection rule, the refractive instrument/protocol, and documentation sufficient to verify cycloplegia at both progression assessments were not retained. A variable labelled cycloplegic_retinoscopy_result is present, but it does not establish the protocol used for both time points. The exact process by which the continuous clinical measurements were rounded or reduced to the three archived values therefore cannot be independently reconstructed from the analytic files.

For the current analysis, the archived binary field fast_progression was operationalized more neutrally as a higher progression category: >=1.50 D over the recorded 12-month interval versus 1.25 D. This threshold is study-specific and should not be interpreted as a universal clinical definition of rapid progression. Because the intervention-stage sample had already been selected from children with rapid progression in the parent study, the 1.25-D group should not be described as clinically ‘slow progression.’

### 2.4 Behavioural exposures and index construction

Four questionnaire-derived composite scores were evaluated: near_work_index = time_school_classes + time_extra_classes + time_self_study; screen_time_index = time_ipad_use + time_phone_use + time_computer_use + time_tv_watching; device_time_index = time_ipad_use + time_phone_use + time_computer_use; and outdoor_index = time_outdoor_sports + time_outdoor_leisure. The reading_risk_index (0–2) was the sum of two derived binary indicators representing close-distance reading and reading while lying in bed. The row-wise formulas were verified against the supplied optimized dataset and codebook.

The source component variables are archived as ordinal numeric codes, but the response anchors corresponding to the full 1–7 coding scale were not available in the supplied codebook. Accordingly, composite scores are reported as archived ordinal-sum indices rather than hours per day, and a one-unit model coefficient denotes one score unit only. The direction and clinical magnitude of these index-unit effects therefore require cautious interpretation. Detailed construction rules and observed ranges are provided in S1 Table; S1 Fig in S2 File.

### 2.5 Covariates and verified category labels

Author-verified labels were applied throughout: sex 1 = Male and 2 = Female; ethnicity 1 = Kinh and 2 = Other; school urbanicity 1 = Urban and 2 = Rural; school region 1 = Lowland/coastal, 2 = Midland, and 3 = Mountainous; school socioeconomic category 1 = Better-off and 2 = Average; baseline myopia severity 1 = Mild, 2 = Moderate, and 3 = High; and parental myopia 1 = No parent, 2 = One parent, and 3=Both parents. Grade was retained as Grades 1–5. Complete variable definitions, coding schemes, and labels for the intervention dataset are provided in S3 File.

School urbanicity and school socioeconomic category were exactly concordant in all 200 records (160 Urban/Better-off and 40 Rural/Average (S2 Table; S2 Fig in S2 File)); they were therefore not entered simultaneously in any model. Urbanicity was used in the primary model and socioeconomic category was treated as a descriptive/sensitivity-equivalent school-context measure.

### 2.6 Missing data

The main behavioural indices, sex, grade, baseline severity, urbanicity, region, socioeconomic category, and outcome were complete in the optimized analytic file. Parental myopia was missing for one participant (0.5%). The primary association analysis therefore used n = 199 complete cases. As a sensitivity analysis, the single missing parental-myopia value was replaced by the modal category and the model was refitted in all 200 participants. Several source clinical or behavioural variables not used in the primary model had substantially greater original missingness, including baseline myopia degree (9.0%), post-cycloplegia myopia degree (18.5%), reading-distance category (54.0%), glasses-use pattern (68.0%), and age at starting glasses (69.5%); these are reported in S3 Table; S3 Fig in S2 File. Outcomes were never imputed [13].

### 2.7 Association analysis and model stability

To address the ratio of sample size to model complexity, the revised primary inferential model was deliberately parsimonious. We fitted logistic regression for the higher progression category and calculated school-clustered robust standard errors. The fixed-effect set comprised device-time index, outdoor index, near-work index, reading-risk index, sex, grade, baseline myopia severity, parental myopia, and school urbanicity. Grade and baseline severity were modelled as ordered one-level increments to conserve degrees of freedom; sex, parental myopia, and urbanicity were categorical. The model estimated 10 predictor coefficients (excluding the intercept) for 99 higher-category outcomes, corresponding to 9.9 outcomes per predictor coefficient. Prespecified interactions from the original analysis were removed from the primary model because the available sample did not support stable estimation of a highly interacted specification.

We report adjusted odds ratios (aORs) with 95% confidence intervals and two-sided p values. Collinearity was assessed using variance-inflation factors. Model stability was examined by refitting the same specification after omitting each school in turn and summarizing the resulting odds-ratio ranges. Given only 10 schools and highly unequal cluster sizes (2–60 students), we did not treat a random-intercept variance or intraclass correlation coefficient as a precisely estimable population parameter. As a sensitivity analysis, we fitted a population-averaged generalized estimating equation (GEE) with exchangeable working correlation.

### 2.8 Cross-school prediction

Prediction was evaluated as an out-of-school transportability test. A core L2-penalized logistic model included sex, grade, baseline severity, parental myopia, and school urbanicity. A behavioural-augmented model added device-time, outdoor, near-work, and reading-risk indices. The primary validation scheme was leave-one-school-out cross-validation, so no student from the held-out school contributed to model fitting. Within each training fold, missing values were imputed from training data and numeric variables were standardized before application to the held-out school. Five-fold GroupKFold by school was used as a sensitivity analysis [14]. Performance was summarized by the area under the receiver-operating-characteristic curve (AUC) and Brier score [15,16]. Because discrimination was expected to be modest and only 10 schools were available, these internally validated metrics were interpreted descriptively rather than as evidence of clinical utility. Calibration plots are provided in the Supplement [17].

### 2.9 Reporting checklists

Completed STROBE and TRIPOD reporting checklists are supplied as Supporting Information (S4 File). TRIPOD items are applied specifically to the prediction component; the association analysis remains observational and is reported under STROBE [11,12,18].

### 2.10 Ethics approval

This secondary analysis used de-identified data from the parent school-based study. Parent-study documentation states that data collection and research use were approved by Council of the University of Medicine and Pharmacy at Ho Chi Minh City (approval No.523/HĐĐĐ-ĐHYD; protocol code 24268-ĐHYD; IRB VN01002/IORG0008603/FWA00023448; approved 27 March 2024) and that written informed consent was obtained from parents or legal guardians. No additional participant contact occurred for this secondary analysis.

## 3. Results

### 3.1 Participant characteristics

The dataset contained 200 students from 10 schools; school cluster sizes ranged from 2 to 60. The higher progression category (>=1.50 D) included 99/200 students (49.5%). Author-verified descriptive labels are used in Table 1. The cohort was 55.5% male and 44.5% female, 99.0% Kinh, and predominantly urban (80.0%). Baseline myopia severity was Mild in 61.0%, Moderate in 34.5%, and High in 4.5%. One parental-myopia value was missing.

**Table 1:** Participant characteristics overall and by recorded 12-month progression category.

Characteristic	Overall (n = 200)	1.25 D (n = 101)	Higher category >=1.50 D (n = 99)
Sex: Male	111 (55.5%)	59 (58.4%)	52 (52.5%)
Sex: Female	89 (44.5%)	42 (41.6%)	47 (47.5%)
Ethnicity: Kinh	198 (99.0%)	101 (100.0%)	97 (98.0%)
Ethnicity: Other	2 (1.0%)	0 (0.0%)	2 (2.0%)
Grade: Grade 1	32 (16.0%)	20 (19.8%)	12 (12.1%)
Grade: Grade 2	38 (19.0%)	18 (17.8%)	20 (20.2%)
Grade: Grade 3	40 (20.0%)	21 (20.8%)	19 (19.2%)
Grade: Grade 4	38 (19.0%)	20 (19.8%)	18 (18.2%)
Grade: Grade 5	52 (26.0%)	22 (21.8%)	30 (30.3%)
Baseline severity: Mild	122 (61.0%)	67 (66.3%)	55 (55.6%)
Baseline severity: Moderate	69 (34.5%)	28 (27.7%)	41 (41.4%)
Baseline severity: High	9 (4.5%)	6 (5.9%)	3 (3.0%)
Parental myopia: No parent	20 (10.0%)	12 (11.9%)	8 (8.1%)
Parental myopia: One parent	59 (29.5%)	30 (29.7%)	29 (29.3%)
Parental myopia: Both parents	120 (60.0%)	58 (57.4%)	62 (62.6%)
Parental myopia: Missing	1 (0.5%)	1 (1.0%)	0 (0.0%)
School urbanicity: Urban	160 (80.0%)	73 (72.3%)	87 (87.9%)
School urbanicity: Rural	40 (20.0%)	28 (27.7%)	12 (12.1%)
School region: Lowland/coastal	141 (70.5%)	68 (67.3%)	73 (73.7%)
School region: Midland	48 (24.0%)	25 (24.8%)	23 (23.2%)
School region: Mountainous	11 (5.5%)	8 (7.9%)	3 (3.0%)
School socioeconomic category: Better-off	160 (80.0%)	73 (72.3%)	87 (87.9%)
School socioeconomic category: Average	40 (20.0%)	28 (27.7%)	12 (12.1%)
Near-work index, median [IQR]	8 [7–10]	8 [7–10]	9 [7–10]
Screen-time index, median [IQR]	16 [10-18.25]	12 [9–17]	16 [11–21]
Device-time index, median [IQR]	11 [7.75-15]	9 [7–15]	15 [9–16]
Outdoor index, median [IQR]	4 [2–8]	4 [2–8]	4 [2–8]

**Note:** Behavioural indices are archived ordinal-sum scores, not hours/day. School socioeconomic category is perfectly concordant with urbanicity and is shown descriptively only.

### 3.2 Outcome distribution and selection context

The archived 12-month progression values were 1.25 D in 101/200 students (50.5%), 1.50 D in 94/200 (47.0%), and 1.75 D in 5/200 (2.5%) (Fig 1A). Thus, 99/200 (49.5%) were assigned to the study-specific higher category of>=1.50 D. Because the intervention-stage sample had been selected from parent-study rapid progressors, the narrow distribution is consistent with strong range restriction. Nevertheless, the supplied archive does not document why only these three values were retained, so the exact discretization/rounding mechanism cannot be verified.

**Fig 1:**
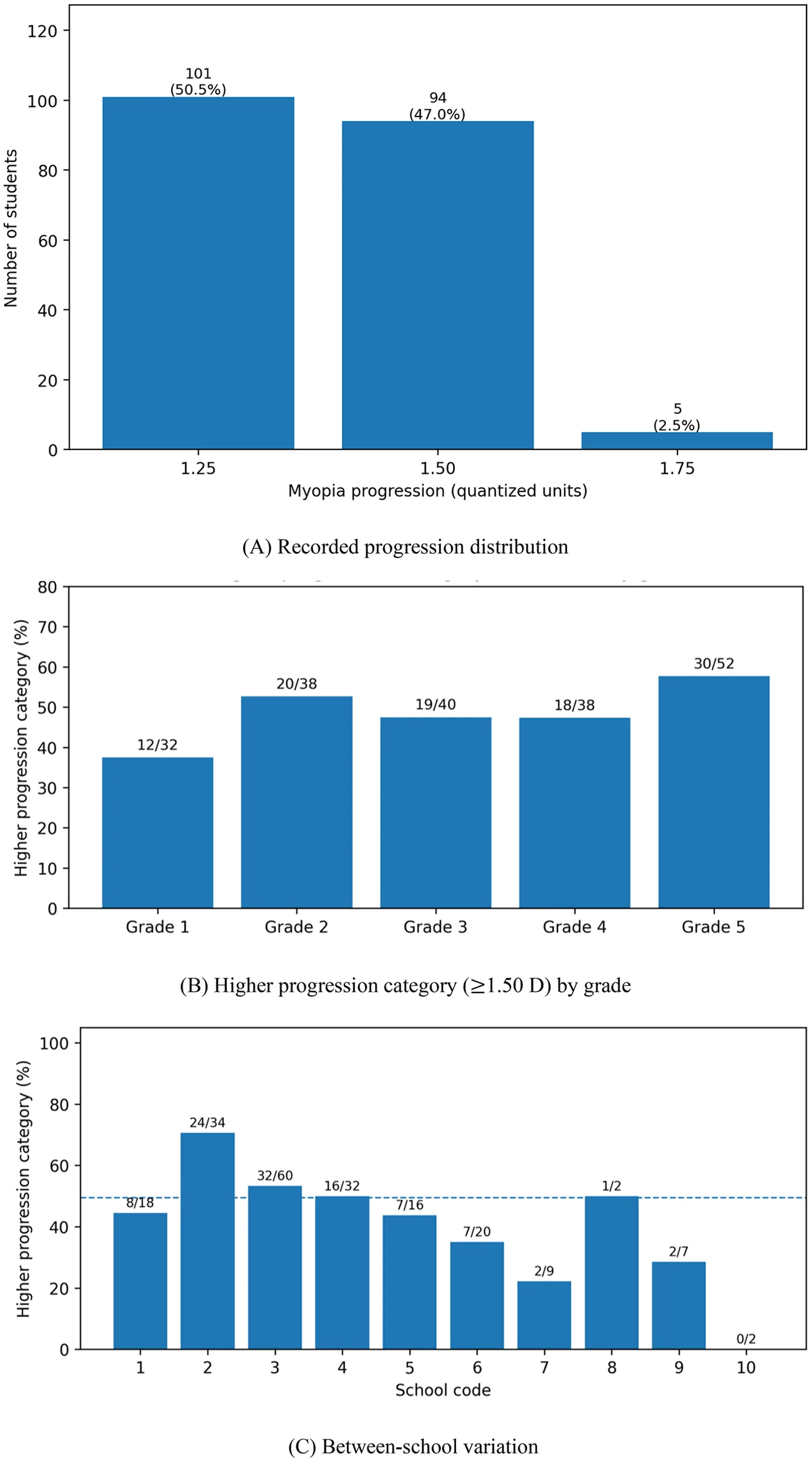
Recorded 12-month progression distribution and heterogeneity. The binary contrast is a study-specific higher progression category (≥1.50 D) versus 1.25 D; it is not a comparison of clinically slow versus fast progressors in an unselected cohort. (A) Recorded progression distribution. (B) Higher progression category (≥1.50 D) by grade. (C) Between-school variation.

The proportion in the higher category ranged from 37.5% in Grade 1 to 57.7% in Grade 5 (Fig 1B). School-specific proportions were highly variable, particularly in schools with very small cluster sizes (Fig 1C). Urban schools had 87/160 (54.4%) students in the higher category versus 12/40 (30.0%) in rural schools. Because urbanicity and socioeconomic category were exactly concordant, this contrast cannot be separated from the corresponding school socioeconomic classification.

### 3.3 Parsimonious cluster-aware association model

The primary complete-case analysis included 199 students and 99 higher-category outcomes. With 10 predictor coefficients excluding the intercept, the model had 9.9 outcomes per predictor coefficient. Variance-inflation factors for predictor terms were modest after excluding the perfectly redundant socioeconomic variable; all non-intercept VIFs were below 3.1 (S8 Table in S2 File).

None of the four behavioural indices showed an independently estimated association with the higher progression category at the conventional 0.05 level after parsimonious adjustment (Table 2; Fig 2). Device-time had aOR 1.074 per score unit (95% CI 0.993–1.161; p = 0.075), outdoor index aOR 1.015 (0.929–1.109; p = 0.738), near-work index aOR 0.972 (0.825–1.145; p = 0.735), and reading-risk index aOR 1.091 (0.799–1.490; p = 0.584). Grade level was associated with the higher category (aOR 1.210 per grade; 95% CI 1.099–1.332; p < 0.001). The rural-versus-urban estimate was imprecise (aOR 0.464; 95% CI 0.181–1.187; p = 0.109).

**Table 2:** Parsimonious association model for the higher recorded 12-month progression category (school-clustered robust standard errors).

Predictor	aOR	95% CI	p value	Interpretation/reference
Female vs Male	1.277	0.801-2.038	0.304	Male reference
One parent vs No parent	1.316	0.245-7.068	0.749	No parent reference
Both parents vs No parent	1.129	0.328-3.882	0.847	No parent reference
Rural vs Urban	0.464	0.181-1.187	0.109	Urban reference
Device-time index (per score unit)	1.074	0.993-1.161	0.075	Per archived score unit
Outdoor index (per score unit)	1.015	0.929-1.109	0.738	Per archived score unit
Near-work index (per score unit)	0.972	0.825-1.145	0.735	Per archived score unit
Reading-risk index (per score unit)	1.091	0.799-1.490	0.584	Per archived score unit
Grade (per level)	1.210	1.099-1.332	<0.001	Per one grade
Baseline severity (per category)	1.102	0.708-1.713	0.668	Per category (Mild- > Moderate- > High)

**Note:** n = 199 complete cases; 99 higher-category outcomes; 10 predictor coefficients excluding the intercept. School socioeconomic category was not entered because it was perfectly concordant with urbanicity.

**Fig 2:**
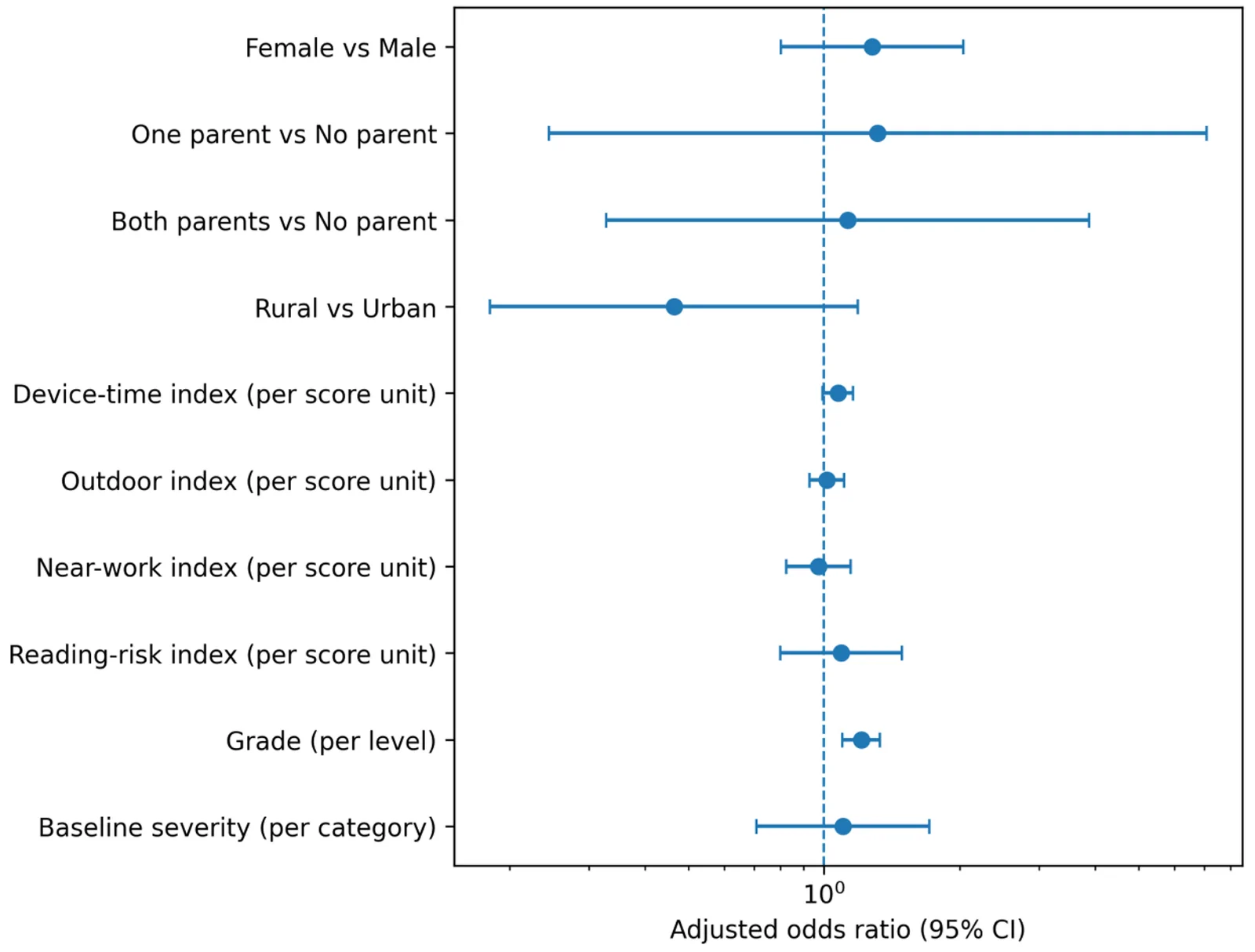
Adjusted odds ratios from the parsimonious cluster-aware association model. Behavioural-index units are archived ordinal-sum score units and cannot be translated into hours/day from the supplied codebook.

### 3.4 Model stability and cluster sensitivity

Leave-one-school-out refitting showed that the behavioural point estimates were not uniformly stable in direction: device-time aORs ranged from 1.062 to 1.122, outdoor from 0.980 to 1.051, near-work from 0.953 to 1.083, and reading-risk from 0.944 to 1.228. The grade estimate remained above 1 in every omitted-school refit (aOR range 1.187–1.280). A GEE exchangeable sensitivity analysis gave a similar overall pattern and an estimated working correlation close to zero; given only 10 unequal clusters, this correlation estimate is descriptive rather than a precise population ICC (S4 and S5 Fig; S4 and S5 Tables in S2 File).

### 3.5 Missing-data sensitivity

All principal behavioural indices and most model covariates were complete; only parental myopia was missing in one record. Replacing that single value with the modal category and refitting all 200 records did not materially alter the results (S6 Table in S2 File). This limited sensitivity analysis was preferred to unnecessary multiple imputation for a single missing covariate value.

### 3.6 Cross-school prediction

Prediction was poor under leave-one-school-out validation (Table 3; Fig 3; S6 Fig in S2 File). The core model achieved AUC 0.540 with Brier score 0.252; adding the four behavioural indices produced AUC 0.570 but a slightly worse Brier score of 0.254. Thus, behavioural augmentation did not yield clinically useful cross-school prediction. Five-fold GroupKFold sensitivity results were similar (AUC 0.534 versus 0.557; S7 Table in S2 File). These values are close to chance discrimination and do not support implementation for screening, referral, or individualized risk stratification.

**Table 3:** Cluster-aware prediction performance.

Validation	Model	AUC	Brier score	Interpretation
Leave-one-school-out	Core	0.540	0.252	Poor discrimination
Leave-one-school-out	Behavioural-augmented	0.570	0.254	Poor discrimination; no useful incremental value

**Fig 3:**
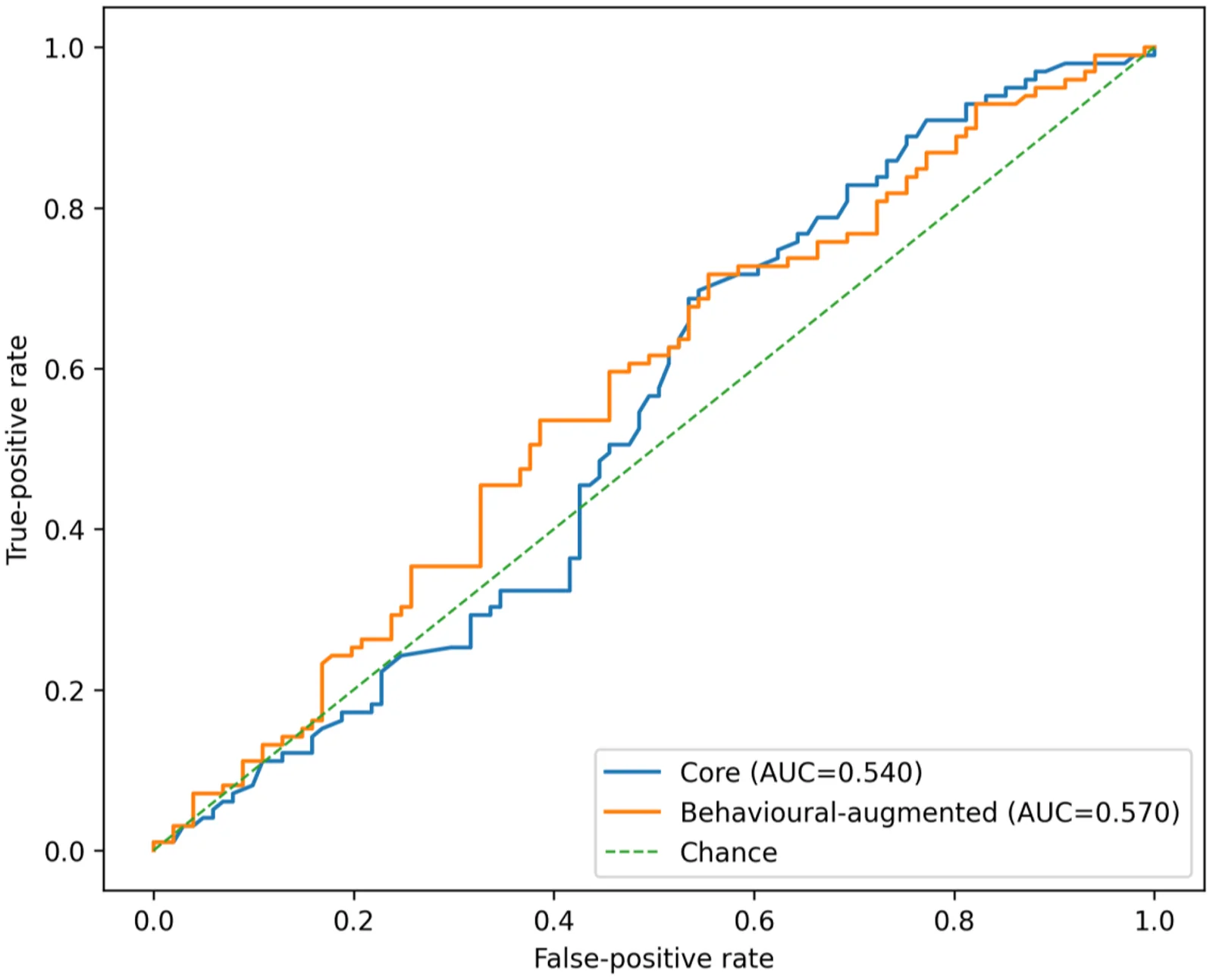
Receiver-operating-characteristic curves under leave-one-school-out validation. Both models showed poor discrimination; the behavioural model should not be interpreted as an implementable prediction tool.

## 4 Discussion

This revision changes the interpretation of the study in several important ways. The 200 students were not an unselected school-based myopia cohort: parent-study documentation indicates that they were purposively selected for an intervention stage after meeting a rapid-progression criterion of ≥ 1.0 D/year. The analytic archive consequently represents a range-restricted high-risk subgroup. Within that subgroup, the three retained 12-month progression values were narrowly distributed, and the exact transformation from the underlying clinical refractions to 1.25, 1.50, and 1.75 D cannot be reconstructed from the supplied analytic files. The present ≥ 1.50-D contrast is therefore a study-specific higher category, not a universally validated definition of fast progression.

After simplifying the association model to match the available sample size, the large behavioural effects reported in the original analysis were not reproduced with stable precision. Device-time showed only weak evidence of a positive association, while outdoor, near-work, and reading-risk scores were compatible with both modest inverse and modest positive associations. Leave-one-school-out refits further showed direction changes for several behavioural coefficients. These findings should therefore be interpreted as exploratory associations measured on archive-specific ordinal-sum scales, not as causal effects or as evidence that a one-unit change corresponds to a clinically meaningful change in hours of exposure.

The result for grade was more stable, with higher grades associated with greater odds of the ≥ 1.50-D category. This may reflect age-related or educational differences, but grade is also a broad proxy for multiple unmeasured factors. The study does not support attributing this association to a specific mechanism. The apparent urban-rural difference likewise cannot be separated from the school socioeconomic classification because the two variables were perfectly concordant in this dataset.

The prediction analysis is a negative validation finding. AUCs of approximately 0.54–0.57 under leave-one-school-out validation, together with Brier scores around 0.25, indicate little transportable individual-level information in the evaluated variables. Adding behavioural questionnaire scores did not produce a useful risk-stratification model. Accordingly, these models should not be used for individual screening, referral decisions, or school deployment. This distinction is important because a variable may show an association in a sample without providing sufficient discrimination or calibration for prediction in a new school.

Only 10 schools were available and cluster sizes were highly unequal (2–60 students). With so few higher-level units, random-effect variances and ICCs can be unstable; the revised analysis therefore uses school-clustered standard errors as the primary approach and GEE/leave-one-school-out refitting as sensitivity analyses rather than placing interpretive weight on a precise ICC. Even cluster-robust standard errors can be imperfect with only 10 clusters, so confidence intervals and p values should be viewed cautiously and replication across more schools is essential.

Missingness in the variables used in the primary model was minimal, but missingness was substantial in several additional clinical variables. More importantly, residual confounding remains a major limitation. The archive did not provide continuous baseline SE for modelling, axial length, parental refractive-error magnitude, sufficiently structured information on atropine use, orthokeratology or other myopia-control interventions, or individual-level socioeconomic measures. Parent-study records indicate that treatment information in the 200-student intervention-stage dataset was largely incomplete. These omissions may be related to both behavioural exposures and progression and therefore preclude causal interpretation.

Measurement provenance is another central limitation. Parent-study documentation establishes a 12-month follow-up and SE-difference framework, but the supplied analytic files do not contain paired eye-level refractions, the eye-selection rule, instrument/examiner details, or enough documentation to establish that progression was based on cycloplegic refraction at both assessments. The presence of a cycloplegia-related field does not resolve this issue. Future studies should prospectively retain eye-specific cycloplegic sphere and cylinder, axial length, exact visit dates, treatment exposure, and a complete data dictionary linking questionnaire categories to physical units.

These limitations do not negate the broader evidence base supporting outdoor time and appropriate visual habits in childhood myopia prevention [5–9,19,20]. Rather, they constrain what can be concluded from this particular selected dataset. The current results are best viewed as a transparent demonstration of the limits of behavioural questionnaires and small-cluster data for individual cross-school prediction, while motivating better-documented longitudinal validation in more representative Vietnamese school populations.

## 5 Conclusion

In this selected high-risk cohort of 200 Vietnamese schoolchildren, the archived 12-month progression outcome was restricted to 1.25, 1.50, and 1.75 D. A parsimonious cluster-aware reanalysis did not identify robust independent associations between the four questionnaire-derived behavioural indices and the ≥ 1.50-D category, although higher grade level was associated with higher odds. Cross-school prediction remained poor and should not be implemented for individual risk stratification. Interpretation is limited by range restriction, only 10 schools, incomplete clinical provenance, unavailable objective ocular/treatment measures, and questionnaire score anchors that cannot be translated into hours/day. Larger prospective studies with standardized cycloplegic refraction, axial length, treatment documentation, objective exposure measurement, and external validation are required.

## Supporting information

S1 File Intervention dataset. De-identified dataset of the 200 participants included in the intervention analysis.
(CSV)

S2 File Supplementary Figures and Tables. This file contains Supplementary Figures S1–S6 and Supplementary Tables S1–S8, including behavioural index distributions, school-level characteristics, missingness assessment, sensitivity analyses, leave-one-school-out analyses, calibration plots, variable definitions, prediction robustness, and collinearity diagnostics.
(DOCX)

S3 File Intervention dataset codebook. Codebook containing variable names, definitions, coding schemes, and labels for the intervention dataset (n = 200).
(CSV)

S4 File STROBE and TRIPOD checklists. Completed STROBE and TRIPOD reporting checklists for the study.
(DOCX)
