# Supplementary material for: Behavioural correlates of higher 12-month myopia progression in a high-risk vietnamese schoolchild cohort and the limits of cross-school prediction
Source: PLoS One. 2026 Oct 6;21(10):e0358487. doi: 10.1371/journal.pone.0358487 (PMC13641380; doi:10.1371/journal.pone.0358487)
Supplement: Supplementary file 1 [file pone.0358487.s002.docx]

**S1 Fig.** Distributions of behavioural indices. Scores are archived ordinal-sum scales; component response/time anchors for the full 1-7 coding scale were not available, so these are not interpreted as hours/day.

**S2 Fig.** Exact concordance between school urbanicity and school socioeconomic category. Urban corresponded to Better-off in all 160 records and Rural to Average in all 40 records.

**S3 Fig.** Selected missingness before analytic recoding. Original missingness is reconstructed from supplied missingness-indicator fields where available.

**S4 Fig.** Leave-one-school-out coefficient stability for selected predictors. Each point is the odds ratio after one of the 10 schools was omitted.

**S5 Fig.** Population-averaged GEE exchangeable sensitivity model. With only 10 unequal school clusters, estimates are interpreted cautiously.

**S6 Fig.** Calibration plots under leave-one-school-out validation for the core and behavioural-augmented prediction models.

**S1 Table.** Variable definitions, verified labels, index construction, and interpretive limits.

| **Variable** | **Missing in optimized** | **Verified definition / labels** | **Construction / range** | **Interpretive note** |
| --- | --- | --- | --- | --- |
| myopia_progression | 0 (0.0%) | Source-recorded 12-month progression values: 1.25, 1.50, 1.75 D | Observed values only | Parent documentation indicates SE change over 12 months; exact discretization and paired eye-level refractions are not retained in this archive. |
| fast_progression | 0 (0.0%) | Existing binary field; revised manuscript calls this higher progression category | 1 if >=1.50 D; 0 if 1.25 D | Study-specific contrast, not a universal rapid-progression threshold; 1.25-D group is not clinically slow in this selected high-risk cohort. |
| sex | 0 (0.0%) | 1=Male; 2=Female | Categorical | Author-verified. |
| ethnicity | 0 (0.0%) | 1=Kinh; 2=Other | Categorical | Author-verified. |
| class_level | 0 (0.0%) | Grades 1-5 | Ordered 1-5 | Modelled per one-grade increment for parsimony. |
| myopia_degree_recode | 0 (0.0%) | 1=Mild; 2=Moderate; 3=High | Ordered 1-3 | Author-verified; modelled per category for parsimony. |
| parental_myopia | 1 (0.5%) | 1=No parent; 2=One parent; 3=Both parents | Categorical | Author-verified. |
| school_urbanicity | 0 (0.0%) | 1=Urban; 2=Rural | Categorical | Author-verified; used in primary model. |
| school_region | 0 (0.0%) | 1=Lowland/coastal; 2=Midland; 3=Mountainous | Categorical | Author-verified; descriptive. |
| school_socioeconomic | 0 (0.0%) | 1=Better-off; 2=Average | Categorical | Author-verified; perfectly concordant with urbanicity, so not fitted jointly. |
| near_work_index | 0 (0.0%) | Archive-specific near-work score | school classes + extra classes + self-study; range 3-19 | Component 1-7 response/time anchors unavailable; not hours/day. |
| screen_time_index | 0 (0.0%) | Archive-specific screen score | iPad/tablet + phone + computer + TV; range 4-28 | Component response/time anchors unavailable; not hours/day. |
| device_time_index | 0 (0.0%) | Archive-specific device score | iPad/tablet + phone + computer; range 3-21 | Component response/time anchors unavailable; not hours/day. |
| outdoor_index | 0 (0.0%) | Archive-specific outdoor score | outdoor sports + outdoor leisure; range 2-14 | Component response/time anchors unavailable; not hours/day. |
| reading_risk_index | 0 (0.0%) | Sum of two derived reading-behaviour indicators | reads_close_yes + reads_bed_yes; range 0-2 | Derived field; original source mapping of the contributing yes/no codes should be retained with the questionnaire in future datasets. |
| cycloplegic_retinoscopy_result | 0 after optimization | Cycloplegia-related source field | Coded source variable | Does not by itself establish cycloplegic refraction at both progression time points. |

**S2 Table.** School urbanicity versus school socioeconomic category.

| **Urbanicity** | **Better-off** | **Average** |
| --- | --- | --- |
| Urban | 160 | 0 |
| Rural | 0 | 40 |

*Cramér’s V = 1.000 (perfect concordance). The two variables contain the same two-group partition in this dataset and were not entered simultaneously in any revised model.*

**S3 Table.** Quantitative missingness summary and analysis handling.

| **Variable** | **Missing n** | **Missing %** | **Used in primary model?** | **Handling** |
| --- | --- | --- | --- | --- |
| Outcome / fast_progression | 0 | 0.0% | Yes | Not imputed |
| near_work_index | 0 | 0.0% | Yes | Complete |
| device_time_index | 0 | 0.0% | Yes | Complete |
| outdoor_index | 0 | 0.0% | Yes | Complete |
| reading_risk_index | 0 | 0.0% | Yes | Complete |
| sex | 0 | 0.0% | Yes | Complete |
| class_level | 0 | 0.0% | Yes | Complete |
| myopia_degree_recode | 0 | 0.0% | Yes | Complete after supplied recoding |
| parental_myopia | 1 | 0.5% | Yes | Primary complete-case n=199; modal-imputation sensitivity n=200 |
| school_urbanicity | 0 | 0.0% | Yes | Complete |
| myopia_degree (original) | 18 | 9.0% | No | Reported for transparency |
| cycloplegic_retinoscopy_result (original) | 10 | 5.0% | No | Reported for transparency |
| myopia_degree_post_cyclo (original) | 37 | 18.5% | No | Reported for transparency |
| reading_distance_category (original) | 108 | 54.0% | No | Reported for transparency |
| glasses_use_pattern (original) | 136 | 68.0% | No | Reported for transparency |
| age_started_wearing_glasses (original) | 139 | 69.5% | No | Reported for transparency |

**S4 Table.** GEE exchangeable sensitivity analysis.

| **Predictor** | **OR** | **95% CI** | **p value** |
| --- | --- | --- | --- |
| Female vs Male | 1.254 | 0.825-1.908 | 0.289 |
| One parent vs No parent | 1.311 | 0.275-6.255 | 0.734 |
| Both parents vs No parent | 1.111 | 0.360-3.432 | 0.854 |
| Rural vs Urban | 0.574 | 0.262-1.258 | 0.166 |
| Device-time index (per score unit) | 1.077 | 0.997-1.164 | 0.059 |
| Outdoor index (per score unit) | 1.013 | 0.932-1.101 | 0.763 |
| Near-work index (per score unit) | 0.999 | 0.880-1.133 | 0.984 |
| Reading-risk index (per score unit) | 1.073 | 0.814-1.414 | 0.619 |
| Grade (per level) | 1.191 | 1.072-1.324 | 0.001 |
| Baseline severity (per category) | 1.205 | 0.819-1.773 | 0.343 |

*Estimated exchangeable working correlation = -0.016. With only 10 unequal clusters, this value is descriptive.*

**S5 Table.** Leave-one-school-out coefficient stability.

| **Predictor** | **Minimum OR** | **Median OR** | **Maximum OR** |
| --- | --- | --- | --- |
| Grade (per level) | 1.187 | 1.205 | 1.280 |
| Device-time index (per score unit) | 1.062 | 1.070 | 1.122 |
| Near-work index (per score unit) | 0.953 | 0.968 | 1.083 |
| Outdoor index (per score unit) | 0.980 | 1.015 | 1.051 |
| Reading-risk index (per score unit) | 0.944 | 1.098 | 1.228 |

**S6 Table.** Sensitivity analysis after modal imputation of the single missing parental-myopia value (n=200).

| **Predictor** | **aOR** | **95% CI** | **p value** |
| --- | --- | --- | --- |
| Female vs Male | 1.296 | 0.801-2.099 | 0.291 |
| One parent vs No parent | 1.314 | 0.243-7.109 | 0.751 |
| Both parents vs No parent | 1.105 | 0.323-3.778 | 0.873 |
| Rural vs Urban | 0.435 | 0.169-1.122 | 0.085 |
| Device-time index (per score unit) | 1.071 | 0.993-1.156 | 0.077 |
| Outdoor index (per score unit) | 1.018 | 0.934-1.110 | 0.687 |
| Near-work index (per score unit) | 0.970 | 0.824-1.141 | 0.711 |
| Reading-risk index (per score unit) | 1.097 | 0.803-1.498 | 0.560 |
| Grade (per level) | 1.203 | 1.100-1.315 | <0.001 |
| Baseline severity (per category) | 1.108 | 0.716-1.715 | 0.646 |

**S7 Table.** Prediction robustness across cluster-aware validation schemes.

| **Validation** | **Model** | **AUC** | **Brier score** |
| --- | --- | --- | --- |
| LOSO | Core | 0.540 | 0.252 |
| LOSO | Behavioural-augmented | 0.570 | 0.254 |
| GroupKFold-5 | Core | 0.534 | 0.254 |
| GroupKFold-5 | Behavioural-augmented | 0.557 | 0.257 |

*Across both validation schemes, discrimination remained poor and Brier scores were approximately 0.25. These models are not suitable for individualized risk stratification.*

**S8 Table.** Collinearity diagnostics for the revised primary model design matrix.

| **Design-matrix term** | **VIF** |
| --- | --- |
| device_time_index | 1.27 |
| outdoor_index | 1.21 |
| near_work_index | 1.37 |
| reading_risk_index | 1.17 |
| class_level | 1.11 |
| myopia_degree_recode | 1.08 |
| sex_2 | 1.05 |
| parental_myopia_2.0 | 2.92 |
| parental_myopia_3.0 | 3.02 |
| school_urbanicity_2 | 1.52 |
