# Supplementary material for: Behavioural correlates of higher 12-month myopia progression in a high-risk vietnamese schoolchild cohort and the limits of cross-school prediction
Source: PLoS One. 2026 Oct 6;21(10):e0358487. doi: 10.1371/journal.pone.0358487 (PMC13641380; doi:10.1371/journal.pone.0358487)
Supplement: Supplementary file 2 [file pone.0358487.s004.docx]

**STROBE crosswalk**

| **Item** | **Concise reporting requirement** | **Location** |
| --- | --- | --- |
| 1 | Title/abstract identify design and provide balanced summary | Title; Abstract |
| 2 | Scientific background and rationale | Introduction |
| 3 | Objectives and prespecified questions | End of Introduction |
| 4 | Key study-design elements | Methods 2.1 |
| 5 | Setting, schools, follow-up context | Methods 2.1 |
| 6 | Participants, selection and eligibility | Methods 2.1-2.2 |
| 7 | Outcomes, exposures, predictors and confounders | Methods 2.3-2.5; Table S1 |
| 8 | Data sources and measurement | Methods 2.3-2.5; Table S1 |
| 9 | Potential sources of bias | Methods 2.1-2.8; Discussion |
| 10 | Study size and model-complexity justification | Methods 2.7; Results 3.3 |
| 11 | Handling of quantitative variables | Methods 2.4, 2.7 |
| 12 | Statistical methods, clustering, missing data, sensitivity analyses | Methods 2.6-2.8; Tables S2-S8 |
| 13 | Participant flow / analytic denominators | Methods 2.2; Results 3.1 |
| 14 | Descriptive data and missingness | Table 1; Table S2 |
| 15 | Outcome data | Results 3.2; Figure 1 |
| 16 | Main adjusted estimates with precision | Results 3.3; Table 2 |
| 17 | Other and sensitivity analyses | Results 3.4-3.6; Supplement |
| 18 | Summary of key results | Discussion opening |
| 19 | Limitations and direction/magnitude of potential bias | Discussion |
| 20 | Cautious overall interpretation | Discussion; Conclusion |
| 21 | Generalisability | Discussion |
| 22 | Funding | Funding statement |

**TRIPOD crosswalk for the prediction component**

| **Item** | **Concise reporting requirement** | **Location** |
| --- | --- | --- |
| 1 | Prediction study identified in title/abstract | Title; Abstract |
| 2 | Background and rationale for prediction question | Introduction |
| 3 | Prediction objective and intended transportability setting | End of Introduction |
| 4 | Data source and setting | Methods 2.1 |
| 5 | Participants and eligibility | Methods 2.2 |
| 6 | Outcome definition and limitations | Methods 2.3 |
| 7 | Predictor definitions and availability | Methods 2.4-2.5; Table S1 |
| 8 | Sample size / complexity considerations | Methods 2.7-2.8 |
| 9 | Missing-data handling within validation folds | Methods 2.6, 2.8 |
| 10 | Model type and predictor handling | Methods 2.8 |
| 11 | Internal validation respecting school clusters | Methods 2.8 |
| 12 | Performance measures | Methods 2.8 |
| 13 | Participant and outcome counts | Results 3.1-3.2 |
| 14 | Model specification / feature sets | Methods 2.8; Table 3 |
| 15 | Model performance | Results 3.6; Table 3; Figure 3; Table S7 |
| 16 | Calibration assessment | Figure S6 |
| 17 | Sensitivity analyses | Table S7 |
| 18 | Interpretation in light of objectives and limitations | Discussion |
| 19 | Limitations of development/validation | Discussion |
| 20 | Potential use and non-implementation statement | Discussion; Conclusion |
| 21 | Supplementary data/code availability statement | Data availability; Supplement |
| 22 | Funding and conflicts | Funding; Competing Interest |
