# Supplementary material for: Behavioural correlates of higher 12-month myopia progression in a high-risk vietnamese schoolchild cohort and the limits of cross-school prediction
Source: PLoS One. 2026 Oct 6;21(10):e0358487. doi: 10.1371/journal.pone.0358487 (PMC13641380; doi:10.1371/journal.pone.0358487)
Supplement: Supplementary file 3 [file pone.0358487.s006.docx]

**Dear Editor and Reviewers,**

We sincerely thank the Editor and Reviewers for their careful and constructive assessment of our manuscript. The comments prompted substantial revisions to the outcome description, statistical analysis, reporting of categorical variables, treatment of clustering and missing data, prediction analyses, and interpretation of the findings.

Most importantly, we have: (1) clarified the 12-month outcome provenance and explicitly acknowledged aspects that cannot be reconstructed from the de-identified analytic archive; (2) replaced the original highly parameterized model with a substantially more parsimonious cluster-aware analysis; (3) removed the simultaneous inclusion of perfectly concordant school-level variables and removed interaction terms from the primary model; (4) added GEE, leave-one-school-out stability, missing-data, and collinearity sensitivity analyses; (5) reframed the prediction component as a negative transportability/validation finding; (6) expanded the discussion of residual confounding and measurement limitations; and (7) provided STROBE and TRIPOD reporting checklists.

The manuscript title, Abstract, Methods, Results, Discussion, Conclusions, tables, figures, and Supporting Information have been revised accordingly.

**Reviewer #1**

**Comment: Several merits to this paper, however, you need to decode certain things, gender, urban area/location etc. So as to enable a clinician understand the paper better. Other than that, this is a very important and relevant paper.**

**Response:** Thank you for this helpful comment and for recognizing the relevance of the study. We agree that presenting categorical variables only as numeric codes substantially reduced clinical readability.

We have now replaced the numeric codes with their verified descriptive labels throughout the manuscript, tables, and Supporting Information. Specifically:

- Sex: 1 = Male, 2 = Female;
- Ethnicity: 1 = Kinh, 2 = Other;
- School urbanicity: 1 = Urban, 2 = Rural;
- School region: 1 = Lowland/coastal, 2 = Midland, 3 = Mountainous;
- School socioeconomic category: 1 = Better-off, 2 = Average;
- Baseline myopia severity: 1 = Mild, 2 = Moderate, 3 = High;
- Parental myopia: 1 = No parent, 2 = One parent, 3 = Both parents.

These definitions are now stated explicitly in Methods, Section 2.5, and descriptive labels are used directly in Table 1, the regression results, and Supplementary Table S1. Numeric codes are retained only where necessary to document the original data structure.

**Changes made:** Methods Section 2.5; Table 1; Table 2; Supplementary Table S1 and related text.

----------

**Reviewer #2**

**Major Comment 1 – Definition and measurement of myopia progression**

**Comment:** The reviewer raised concerns regarding how myopia progression was determined, including the follow-up interval, calculation of refractive change, cycloplegic measurement, and the unusual presence of only three progression values (1.25, 1.50, and 1.75 D).

**Response:** We agree that the original manuscript did not provide sufficient information to interpret the progression outcome appropriately.

We revisited the parent-study documentation and the supplied analytic archive. Parent-study documentation indicates that participants were re-examined after approximately 12 months and that progression was calculated from the change in spherical-equivalent refraction over this interval. The 200 participants included in the present analysis had been purposively selected for the intervention stage of the parent study after meeting its rapid-progression criterion of at least 1.0 D/year.

However, the de-identified analytic archive available for this secondary analysis retains only three progression values—1.25 D, 1.50 D, and 1.75 D—and does not contain paired eye-level sphere/cylinder measurements, continuous baseline and follow-up SE values, an eye-selection rule, examination dates, or sufficiently detailed documentation to independently reconstruct the exact discretization mechanism. Although a cycloplegia-related variable is present, the archive is also insufficient to establish that progression was determined from cycloplegic refraction at both examinations.

We have therefore deliberately avoided filling these gaps by assumption. Instead, we substantially revised the manuscript to make the measurement provenance transparent.

In particular, the binary endpoint formerly described as “fast progression” is now referred to more neutrally as a study-specific higher progression category (≥1.50 D over the recorded 12-month interval versus 1.25 D). We explicitly state that this threshold should not be interpreted as a universal clinical definition of rapid progression and that the 1.25-D group should not be considered a clinically slow-progression group, because all participants arose from a preselected high-risk cohort.

The title, Abstract, Methods, Discussion, and Conclusions have been revised to reflect this more cautious interpretation.

**Changes made:** Title; Abstract; Methods Sections 2.1–2.3; Discussion; Conclusions; Supplementary Table S1.

**Major Comment 2 – Model complexity and risk of overfitting**

**Comment:** The reviewer considered the original multivariable model excessively complex relative to the sample size and number of events, particularly because of the large number of covariates and interaction terms.

**Response:** We agree and have substantially restructured the primary association analysis.

The original highly parameterized specification and interaction terms are no longer used as the primary model. The revised primary analysis is a parsimonious logistic regression with school-clustered robust standard errors. It includes four prespecified behavioural indices together with a limited covariate set: sex, grade, baseline myopia severity, parental myopia, and school urbanicity.

Grade and baseline severity are modelled as ordered one-level increments to conserve degrees of freedom. The interaction terms from the original analysis have been removed from the primary model. In addition, school urbanicity and school socioeconomic category are no longer fitted simultaneously because our re-examination demonstrated that they are perfectly concordant in this dataset.

The revised model estimates 10 predictor coefficients for 99 higher-progression outcomes, corresponding to approximately 9.9 outcomes per predictor coefficient, compared with the considerably more highly parameterized original analysis.

We also added several stability checks:

- variance-inflation-factor assessment;
- leave-one-school-out refitting;
- GEE with exchangeable working correlation;
- sensitivity analysis for the single missing parental-myopia observation.

Importantly, the conclusions were updated according to the revised analysis rather than attempting to preserve the original results. None of the four behavioural indices showed a robust independent association in the revised primary model:

- device-time index: aOR 1.074, 95% CI 0.993–1.161;
- outdoor index: aOR 1.015, 95% CI 0.929–1.109;
- near-work index: aOR 0.972, 95% CI 0.825–1.145;
- reading-risk index: aOR 1.091, 95% CI 0.799–1.490.

Grade level remained associated with the higher progression category (aOR 1.210 per grade, 95% CI 1.099–1.332).

**Changes made:** Methods Section 2.7; Results and revised Table 2; Supplementary Tables S4–S6 and S8; Supplementary Figures S4–S5; Discussion and Conclusions.

**Major Comment 3 – Only 10 school clusters and uncertainty in multilevel estimates**

**Comment:** The reviewer noted that only 10 schools were available, making random-effect variance and ICC estimation potentially unstable.

**Response:** We agree. We have revised both the analysis and interpretation to avoid placing undue emphasis on a random-intercept variance or ICC estimated from only 10 schools with highly unequal cluster sizes.

School sizes ranged from 2 to 60 students, making precise estimation of higher-level variance particularly uncertain. Consequently, the revised primary model uses school-clustered robust standard errors rather than interpreting a random-intercept variance or ICC as a precisely estimated population parameter.

We additionally performed a population-averaged GEE exchangeable sensitivity analysis and leave-one-school-out refitting to examine whether the results were dependent on individual schools.

The Discussion now explicitly states that even cluster-robust inference should be interpreted cautiously with only 10 clusters, and we no longer use the original ICC estimate as evidence of the magnitude or absence of between-school heterogeneity.

**Changes made:** Methods Section 2.7; Results; Supplementary Tables S4–S5; Supplementary Figures S4–S5; Discussion.

**Major Comment 4 – Poor predictive performance and interpretation of the prediction model**

**Comment:** The reviewer emphasized that the prediction models performed poorly and questioned whether they provide clinically useful prediction.

**Response:** We agree fully and have substantially reframed this component of the manuscript.

The revised manuscript no longer presents the prediction analysis as development of an implementable clinical prediction tool. Instead, it is explicitly framed as a test of cross-school transportability: whether the available demographic, school-context, and behavioural information can discriminate the higher progression category when applied to students from an unseen school.

The primary validation now uses leave-one-school-out cross-validation. Performance remained poor:

- Core model: AUC **=** 0.540, Brier score = 0.252;
- Behavioural-augmented model: AUC = 0.570, Brier score = 0.254.

Five-fold GroupKFold analysis by school produced similarly limited discrimination.

We therefore state explicitly that these models should not be used for individualized screening, referral, clinical risk stratification, or treatment decisions.

Analyses that could encourage overinterpretation of a poorly performing model, including the previous SHAP-focused interpretation, have been removed. The Abstract, Discussion, and Conclusions now present poor prediction as an important negative validation finding rather than attempting to infer clinical utility from near-chance discrimination.

**Changes made:** Abstract; Methods Section 2.8; Results and Table 3; Supplementary Table S7 and Figure S6; Discussion; Conclusions.

**Major Comment 5 – Missing data**

**Comment:** The reviewer requested clearer reporting of missing data and the methods used to handle missingness.

**Response:** Thank you. We have completely revised the missing-data description and now report missingness quantitatively rather than relying on a generic imputation statement.

All variables used in the primary model were complete except parental myopia, which was missing for only one participant (0.5%). The primary association analysis therefore uses 199 complete cases. Outcomes were not imputed.

As a sensitivity analysis, the single missing parental-myopia observation was replaced by the modal category and the model was refitted using all 200 participants. The substantive conclusions were unchanged.

We also report missingness for additional source variables that were not included in the primary model, including:

- original myopia degree: 9.0%;
- post-cycloplegia myopia degree: 18.5%;
- reading-distance category: 54.0%;
- glasses-use pattern: 68.0%;
- age at starting glasses: 69.5%.

The previous generic multiple-imputation description was removed because it did not accurately represent the revised primary analysis.

A complete quantitative missingness table and analysis handling are now provided in Supplementary Table S2.

**Changes made:** Methods Section 2.6; Supplementary Table S2; Supplementary Figure S1; sensitivity analysis in Supplementary Table S6.

**Major Comment 6 – Residual confounding and causal interpretation**

**Comment:** The reviewer noted that several clinically important factors were unavailable and that the reported associations could be affected by residual confounding.

**Response:** We agree and have substantially strengthened this limitation.

The revised Discussion now explicitly identifies several clinically important variables that were unavailable or insufficiently characterized in the analytic archive, including continuous baseline spherical equivalent, axial length, magnitude of parental refractive error, detailed information on low-dose atropine, orthokeratology and other myopia-control interventions, individual-level socioeconomic indicators, and objective measurements of behavioural exposures.

These factors could be associated with both recorded behaviours and subsequent refractive progression and therefore represent potential sources of residual confounding.

We have consequently removed or softened causal language throughout the manuscript. The observed estimates are now consistently described as associations/correlates, rather than protective effects, risk effects, or evidence supporting causal behavioural interventions.

The Conclusions now explicitly state that the findings should not be used to make causal behavioural claims.

**Changes made:** Abstract; Results interpretation; Discussion; Limitations; Conclusions.

**Minor Comments**

**Minor Comment 1 – Construction and interpretation of behavioural indices**

**Comment:** The reviewer requested clearer definitions of the near-work, screen-time, device-time, outdoor, and reading-behaviour indices.

**Response:** We agree. We now provide the exact construction of each composite index:

- near_work_index = school classes + extra classes + self-study;
- screen_time_index = iPad/tablet use + phone use + computer use + television viewing;
- device_time_index = iPad/tablet use + phone use + computer use;
- outdoor_index = outdoor sports + outdoor leisure;
- reading_risk_index = close-reading indicator + reading-in-bed indicator.

During this audit, we also determined that the de-identified archive does not contain the original response anchors corresponding to the full 1–7 coding scale of the component variables. We have therefore avoided interpreting these scores as hours/day.

They are now described consistently as archive-specific ordinal-sum scores, and a one-unit odds ratio refers only to one score unit. This limitation is stated explicitly in the Methods and Supplementary Material.

**Changes made:** Methods Section 2.4; Supplementary Table S1; Supplementary Figure S2.

**Minor Comment 2 – Numeric category labels**

**Comment:** The reviewer requested that numerical categories be decoded into clinically understandable labels.

**Response:** We agree and have implemented this throughout the manuscript. The verified labels for sex, ethnicity, urbanicity, geographic region, socioeconomic category, baseline myopia severity, and parental myopia are now presented directly rather than as codes 1/2/3.

For example, Table 1 now reports “Male/Female,” “Urban/Rural,” “Lowland/coastal/Midland/Mountainous,” and “No parent/One parent/Both parents” instead of numerical categories.

A complete mapping is provided in Methods Section 2.5 and Supplementary Table S1.

**Changes made:** Methods Section 2.5; Table 1; Table 2; Supplementary Table S1.

**Minor Comment 3 – School urbanicity and socioeconomic status**

**Comment:** The reviewer questioned the inclusion and interpretation of multiple school-context variables.

**Response:** Thank you. During the revision we formally examined these variables and found that school urbanicity and school socioeconomic category were perfectly concordant in all 200 observations:

- 160 students were classified as both Urban and Better-off;
- 40 students were classified as both Rural and Average;
- no discordant observations were present.

The corresponding Cramér’s V is 1.000.

Because these variables contain exactly the same two-group partition in this dataset, including them simultaneously would be redundant and potentially destabilize the model.

We therefore use school urbanicity only in the revised primary model, while socioeconomic category is retained for descriptive reporting. Their exact concordance is explicitly documented in Supplementary Table S3 and Supplementary Figure S3.

**Changes made:** Methods Sections 2.5 and 2.7; Table 1; Supplementary Table S3 and Figure S3.

**Minor Comment 4 – STROBE and TRIPOD reporting**

**Comment:** The reviewer requested reporting according to appropriate observational and prediction-model reporting guidelines.

**Response:** We agree. The revised manuscript is explicitly aligned with STROBE for the observational association analysis and TRIPOD for the prediction component.

Completed STROBE and TRIPOD reporting checklists have been prepared as Supporting Information. We also clarify in the Methods that TRIPOD applies specifically to the cross-school prediction component, whereas the primary association analysis is reported according to STROBE principles.

**Changes made:** Introduction; Methods Sections 2.1 and 2.9; separate STROBE and TRIPOD Supporting Information checklists.

--- THE END ---
