# Supplementary material for: Behavioural correlates of higher 12-month myopia progression in a high-risk vietnamese schoolchild cohort and the limits of cross-school prediction
Source: PLoS One. 2026 Oct 6;21(10):e0358487. doi: 10.1371/journal.pone.0358487 (PMC13641380; doi:10.1371/journal.pone.0358487)
Supplement: Peer review history

# Peer review: Decision Letter 0

**DOI:** 10.1371/journal.pone.0358487.r001

**Authors:** Andrzej Grzybowski

21 Jun 2026

Dear Dr. Nguyen,

Thank you for submitting your manuscript to PLOS ONE. After careful consideration, we feel that it has merit but does not fully meet PLOS ONE’s publication criteria as it currently stands. Therefore, we invite you to submit a revised version of the manuscript that addresses the points raised during the review process.

Please submit your revised manuscript by Aug 05 2026 11:59PM. If you will need more time than this to complete your revisions, please reply to this message or contact the journal office at plosone@plos.org. When you're ready to submit your revision, log on to https://www.editorialmanager.com/pone/ and select the 'Submissions Needing Revision' folder to locate your manuscript file.

- A letter that responds to each point raised by the academic editor and reviewer(s). You should upload this letter as a separate file labeled 'Response to Reviewers'.
- A marked-up copy of your manuscript that highlights changes made to the original version. You should upload this as a separate file labeled 'Revised Manuscript with Track Changes'.
- An unmarked version of your revised paper without tracked changes. You should upload this as a separate file labeled 'Manuscript'.

If you would like to make changes to your financial disclosure, please include your updated statement in your cover letter. Guidelines for resubmitting your figure files are available below the reviewer comments at the end of this letter.

If applicable, we recommend that you deposit your laboratory protocols in protocols.io to enhance the reproducibility of your results. Protocols.io assigns your protocol its own identifier (DOI) so that it can be cited independently in the future. For instructions see: https://journals.plos.org/plosone/s/submission-guidelines#loc-laboratory-protocols. Additionally, PLOS ONE offers an option for publishing peer-reviewed Lab Protocol articles, which describe protocols hosted on protocols.io. Read more information on sharing protocols at https://plos.org/protocols?utm_medium=editorial-email&utm_source=authorletters&utm_campaign=protocols.

As the corresponding author, your ORCID iD is verified in the submission system and will appear in the published article. PLOS supports the use of ORCID, and we encourage all coauthors to register for an ORCID iD and use it as well. Please encourage your coauthors to verify their ORCID iD within the submission system before final acceptance, as unverified ORCID iDs will not appear in the published article. *Only* the individual author can complete the verification step; PLOS staff *cannot* verify ORCID iDs on behalf of authors.

We look forward to receiving your revised manuscript.

Kind regards,

Andrzej Grzybowski

Academic Editor

PLOS One

Journal requirements:

When submitting your revision, we need you to address these additional requirements.

1. Please ensure that your manuscript meets PLOS ONE's style requirements, including those for file naming. The PLOS ONE style templates can be found at

https://journals.plos.org/plosone/s/file?id=wjVg/PLOSOne_formatting_sample_main_body.pdf and

https://journals.plos.org/plosone/s/file?id=ba62/PLOSOne_formatting_sample_title_authors_affiliations.pdf

2. In the online submission form you indicate that your data is not available for proprietary reasons and have provided a contact point for accessing this data. Please note that your current contact point is a co-author on this manuscript. According to our Data Policy, the contact point must not be an author on the manuscript and must be an institutional contact, ideally not an individual. Please revise your data statement to a non-author institutional point of contact, such as a data access or ethics committee, and send this to us via return email. Please also include contact information for the third party organization, and please include the full citation of where the data can be found.

3. Your ethics statement should only appear in the Methods section of your manuscript. If your ethics statement is written in any section besides the Methods, please move it to the Methods section and delete it from any other section. Please ensure that your ethics statement is included in your manuscript, as the ethics statement entered into the online submission form will not be published alongside your manuscript.

4. Please include captions for your Supporting Information files at the end of your manuscript, and update any in-text citations to match accordingly. Please see our Supporting Information guidelines for more information: http://journals.plos.org/plosone/s/supporting-information.

If the reviewer comments include a recommendation to cite specific previously published works, please review and evaluate these publications to determine whether they are relevant and should be cited. There is no requirement to cite these works unless the editor has indicated otherwise.

[Note: HTML markup is below. Please do not edit.]

Reviewers' comments:

Reviewer's Responses to Questions

**Comments to the Author**

1. Is the manuscript technically sound, and do the data support the conclusions?

Reviewer #1: Yes

Reviewer #2: Partly

**********

2. Has the statistical analysis been performed appropriately and rigorously?

Reviewer #1: Yes

Reviewer #2: No

**********

3. Have the authors made all data underlying the findings in their manuscript fully available?

The PLOS Data policy

Reviewer #1: No

Reviewer #2: No

**********

4. Is the manuscript presented in an intelligible fashion and written in standard English?

Reviewer #1: Yes

Reviewer #2: Yes

**********

Reviewer #1: Several merits to this paper, however, you need to decode certain things, gender, urban area/location etc. So as to enable a clinician understand the paper better.

Other than that, this is a very important and relevant paper.

Reviewer #2: We would like to thank the authors for their manuscript that investigates behavioural correlates of fast myopia progression in Vietnamese schoolchildren using multilevel modelling and cluster-aware prediction approaches. The study addresses an important public health issue. The manuscript is generally well written. However, several critical issues limit confidence in the findings and their interpretation.

Major Comments

1. Outcome definition and clinical validity

The primary outcome appears unusual. The manuscript states that the observed values of "myopia_progression" were limited to only three categories (1.25, 1.50, and 1.75 D), with 97.5% of observations concentrated in only two categories. The origin of these values is unclear.

• How was progression measured?

• What was the exact follow-up duration?

• Were refractive measurements cycloplegic?

• Why are progression values restricted to only three discrete levels?

Because the entire analysis depends on this outcome variable, the measurement process must be described in substantially greater detail.

2. Sample size versus model complexity

The study includes only 200 participants clustered within 10 schools, with 99 events.

The mixed-effects models simultaneously include:

• multiple behavioural indices,

• demographic covariates,

• parental myopia,

• school-level variables,

• interaction terms,

• random school effects.

This appears potentially overparameterized relative to the available sample size.

The authors should:

• provide justification for model complexity,

• report the number of parameters estimated,

• discuss potential overfitting,

• provide model stability diagnostics.

3. School-level clustering may be inadequately estimated

Only 10 schools were included.

Estimation of random effects and ICCs can be unstable with a small number of clusters.

The authors should discuss:

• the limitations of estimating random intercepts with only 10 clusters

4. Prediction analyses demonstrate essentially no predictive utility

The central predictive results show:

• AUC = 0.524 (core model)

• AUC = 0.514 (behavioural model)

These values are effectively equivalent to random classification.

Despite this, substantial attention is devoted to prediction modelling.

The manuscript should more clearly emphasize that:

• the predictive models failed,

• behavioural questionnaires alone were insufficient for risk prediction,

• these findings argue against implementation of such models in practice.

Currently, parts of the discussion remain overly optimistic given the observed predictive performance.

5. Missing data handling requires clarification

The manuscript describes:

• single imputation,

• missing-indicator approaches,

• multiple imputation sensitivity analyses.

However, no quantitative summary of missingness is provided.

The authors should report:

• missingness percentages for all variables,

• variables requiring imputation,

• number of imputed observations,

• results of sensitivity analyses.

Without this information, the robustness of the findings cannot be evaluated.

6. Potential residual confounding

Important predictors of myopia progression appear unavailable, including:

• axial length,

• baseline spherical equivalent refraction (continuous),

• parental refractive error magnitude,

• atropine use,

• orthokeratology or myopia-control interventions,

• socioeconomic indicators beyond school-level coding.

These omissions should be discussed more explicitly as limitations.

Minor Comments

1.. Clarify coding of indices

Several variables are difficult to interpret:

• near_work_index

• screen_time_index

• device_time_index

• outdoor_index

• reading_risk_index

The exact construction of these indices should be described in greater detail, ideally in a supplementary appendix.

2. Clarify school socioeconomic and urbanicity coding

Variables such as:

• school_urbanicity (code 1 vs 2)

• school_socioeconomic (code 1 vs 2)

are reported without meaningful labels.

Please replace numeric coding with descriptive terminology throughout the manuscript and tables.

3. Figure and table presentation

Table 1 is informative but somewhat difficult to interpret because variable coding is presented numerically rather than descriptively.

Consider replacing coded categories with meaningful labels.

4. Reporting standards

The manuscript cites STROBE and TRIPOD. A completed STROBE checklist and TRIPOD checklist should be included as supplementary material.

The manuscript addresses an important topic and employs several modern analytical approaches. However, substantial clarification is needed regarding outcome measurement, model specification, missing-data handling, cluster-level inference, and interpretation of predictive performance before the conclusions can be fully supported.

**********

what does this mean?). If published, this will include your full peer review and any attached files.

If you choose “no”, your identity will remain anonymous but your review may still be made public.

**Do you want your identity to be public for this peer review?** For information about this choice, including consent withdrawal, please see our Privacy Policy

Reviewer #1: **Yes:**  Obinwanne Chukwuemeka Junior

Reviewer #2: No

**********

[NOTE: If reviewer comments were submitted as an attachment file, they will be attached to this email and accessible via the submission site. Please log into your account, locate the manuscript record, and check for the action link "View Attachments". If this link does not appear, there are no attachment files.]

To ensure your figures meet our technical requirements, please review our figure guidelines: https://journals.plos.org/plosone/s/figures

You may also use PLOS’s free figure tool, NAAS, to help you prepare publication quality figures: https://journals.plos.org/plosone/s/figures#loc-tools-for-figure-preparation.

NAAS will assess whether your figures meet our technical requirements by comparing each figure against our figure specifications.

# Author comment: Author response to Decision Letter 1

**DOI:** 10.1371/journal.pone.0358487.r002

5 Aug 2026

Dear Editor and Reviewers,

We sincerely thank the Editor and Reviewers for their careful and constructive assessment of our manuscript. The comments prompted substantial revisions to the outcome description, statistical analysis, reporting of categorical variables, treatment of clustering and missing data, prediction analyses, and interpretation of the findings.

Most importantly, we have: (1) clarified the 12-month outcome provenance and explicitly acknowledged aspects that cannot be reconstructed from the de-identified analytic archive; (2) replaced the original highly parameterized model with a substantially more parsimonious cluster-aware analysis; (3) removed the simultaneous inclusion of perfectly concordant school-level variables and removed interaction terms from the primary model; (4) added GEE, leave-one-school-out stability, missing-data, and collinearity sensitivity analyses; (5) reframed the prediction component as a negative transportability/validation finding; (6) expanded the discussion of residual confounding and measurement limitations; and (7) provided STROBE and TRIPOD reporting checklists.

The manuscript title, Abstract, Methods, Results, Discussion, Conclusions, tables, figures, and Supporting Information have been revised accordingly.

Reviewer #1

Comment: Several merits to this paper, however, you need to decode certain things, gender, urban area/location etc. So as to enable a clinician understand the paper better. Other than that, this is a very important and relevant paper.

Response: Thank you for this helpful comment and for recognizing the relevance of the study. We agree that presenting categorical variables only as numeric codes substantially reduced clinical readability.

We have now replaced the numeric codes with their verified descriptive labels throughout the manuscript, tables, and Supporting Information. Specifically:

• Sex: 1 = Male, 2 = Female;

• Ethnicity: 1 = Kinh, 2 = Other;

• School urbanicity: 1 = Urban, 2 = Rural;

• School region: 1 = Lowland/coastal, 2 = Midland, 3 = Mountainous;

• School socioeconomic category: 1 = Better-off, 2 = Average;

• Baseline myopia severity: 1 = Mild, 2 = Moderate, 3 = High;

• Parental myopia: 1 = No parent, 2 = One parent, 3 = Both parents.

These definitions are now stated explicitly in Methods, Section 2.5, and descriptive labels are used directly in Table 1, the regression results, and Supplementary Table S1. Numeric codes are retained only where necessary to document the original data structure.

Changes made: Methods Section 2.5; Table 1; Table 2; Supplementary Table S1 and related text.

----------

Reviewer #2

Major Comment 1 – Definition and measurement of myopia progression

Comment: The reviewer raised concerns regarding how myopia progression was determined, including the follow-up interval, calculation of refractive change, cycloplegic measurement, and the unusual presence of only three progression values (1.25, 1.50, and 1.75 D).

Response: We agree that the original manuscript did not provide sufficient information to interpret the progression outcome appropriately.

We revisited the parent-study documentation and the supplied analytic archive. Parent-study documentation indicates that participants were re-examined after approximately 12 months and that progression was calculated from the change in spherical-equivalent refraction over this interval. The 200 participants included in the present analysis had been purposively selected for the intervention stage of the parent study after meeting its rapid-progression criterion of at least 1.0 D/year.

However, the de-identified analytic archive available for this secondary analysis retains only three progression values—1.25 D, 1.50 D, and 1.75 D—and does not contain paired eye-level sphere/cylinder measurements, continuous baseline and follow-up SE values, an eye-selection rule, examination dates, or sufficiently detailed documentation to independently reconstruct the exact discretization mechanism. Although a cycloplegia-related variable is present, the archive is also insufficient to establish that progression was determined from cycloplegic refraction at both examinations.

We have therefore deliberately avoided filling these gaps by assumption. Instead, we substantially revised the manuscript to make the measurement provenance transparent.

In particular, the binary endpoint formerly described as “fast progression” is now referred to more neutrally as a study-specific higher progression category (≥1.50 D over the recorded 12-month interval versus 1.25 D). We explicitly state that this threshold should not be interpreted as a universal clinical definition of rapid progression and that the 1.25-D group should not be considered a clinically slow-progression group, because all participants arose from a preselected high-risk cohort.

The title, Abstract, Methods, Discussion, and Conclusions have been revised to reflect this more cautious interpretation.

Changes made: Title; Abstract; Methods Sections 2.1–2.3; Discussion; Conclusions; Supplementary Table S1.

Major Comment 2 – Model complexity and risk of overfitting

Comment: The reviewer considered the original multivariable model excessively complex relative to the sample size and number of events, particularly because of the large number of covariates and interaction terms.

Response: We agree and have substantially restructured the primary association analysis.

The original highly parameterized specification and interaction terms are no longer used as the primary model. The revised primary analysis is a parsimonious logistic regression with school-clustered robust standard errors. It includes four prespecified behavioural indices together with a limited covariate set: sex, grade, baseline myopia severity, parental myopia, and school urbanicity.

Grade and baseline severity are modelled as ordered one-level increments to conserve degrees of freedom. The interaction terms from the original analysis have been removed from the primary model. In addition, school urbanicity and school socioeconomic category are no longer fitted simultaneously because our re-examination demonstrated that they are perfectly concordant in this dataset.

The revised model estimates 10 predictor coefficients for 99 higher-progression outcomes, corresponding to approximately 9.9 outcomes per predictor coefficient, compared with the considerably more highly parameterized original analysis.

We also added several stability checks:

• variance-inflation-factor assessment;

• leave-one-school-out refitting;

• GEE with exchangeable working correlation;

• sensitivity analysis for the single missing parental-myopia observation.

Importantly, the conclusions were updated according to the revised analysis rather than attempting to preserve the original results. None of the four behavioural indices showed a robust independent association in the revised primary model:

• device-time index: aOR 1.074, 95% CI 0.993–1.161;

• outdoor index: aOR 1.015, 95% CI 0.929–1.109;

• near-work index: aOR 0.972, 95% CI 0.825–1.145;

• reading-risk index: aOR 1.091, 95% CI 0.799–1.490.

Grade level remained associated with the higher progression category (aOR 1.210 per grade, 95% CI 1.099–1.332).

Changes made: Methods Section 2.7; Results and revised Table 2; Supplementary Tables S4–S6 and S8; Supplementary Figures S4–S5; Discussion and Conclusions.

Major Comment 3 – Only 10 school clusters and uncertainty in multilevel estimates

Comment: The reviewer noted that only 10 schools were available, making random-effect variance and ICC estimation potentially unstable.

Response: We agree. We have revised both the analysis and interpretation to avoid placing undue emphasis on a random-intercept variance or ICC estimated from only 10 schools with highly unequal cluster sizes.

School sizes ranged from 2 to 60 students, making precise estimation of higher-level variance particularly uncertain. Consequently, the revised primary model uses school-clustered robust standard errors rather than interpreting a random-intercept variance or ICC as a precisely estimated population parameter.

We additionally performed a population-averaged GEE exchangeable sensitivity analysis and leave-one-school-out refitting to examine whether the results were dependent on individual schools.

The Discussion now explicitly states that even cluster-robust inference should be interpreted cautiously with only 10 clusters, and we no longer use the original ICC estimate as evidence of the magnitude or absence of between-school heterogeneity.

Changes made: Methods Section 2.7; Results; Supplementary Tables S4–S5; Supplementary Figures S4–S5; Discussion.

Major Comment 4 – Poor predictive performance and interpretation of the prediction model

Comment: The reviewer emphasized that the prediction models performed poorly and questioned whether they provide clinically useful prediction.

Response: We agree fully and have substantially reframed this component of the manuscript.

The revised manuscript no longer presents the prediction analysis as development of an implementable clinical prediction tool. Instead, it is explicitly framed as a test of cross-school transportability: whether the available demographic, school-context, and behavioural information can discriminate the higher progression category when applied to students from an unseen school.

The primary validation now uses leave-one-school-out cross-validation. Performance remained poor:

• Core model: AUC = 0.540, Brier score = 0.252;

• Behavioural-augmented model: AUC = 0.570, Brier score = 0.254.

Five-fold GroupKFold analysis by school produced similarly limited discrimination.

We therefore state explicitly that these models should not be used for individualized screening, referral, clinical risk stratification, or treatment decisions.

Analyses that could encourage overinterpretation of a poorly performing model, including the previous SHAP-focused interpretation, have been removed. The Abstract, Discussion, and Conclusions now present poor prediction as an important negative validation finding rather than attempting to infer clinical utility from near-chance discrimination.

Changes made: Abstract; Methods Section 2.8; Results and Table 3; Supplementary Table S7 and Figure S6; Discussion; Conclusions.

Major Comment 5 – Missing data

Comment: The reviewer requested clearer reporting of missing data and the methods used to handle missingness.

Response: Thank you. We have completely revised the missing-data description and now report missingness quantitatively rather than relying on a generic imputation statement.

All variables used in the primary model were complete except parental myopia, which was missing for only one participant (0.5%). The primary association analysis therefore uses 199 complete cases. Outcomes were not imputed.

As a sensitivity analysis, the single missing parental-myopia observation was replaced by the modal category and the model was refitted using all 200 participants. The substantive conclusions were unchanged.

We also report missingness for additional source variables that were not included in the primary model, including:

• original myopia degree: 9.0%;

• post-cycloplegia myopia degree: 18.5%;

• reading-distance category: 54.0%;

• glasses-use pattern: 68.0%;

• age at starting glasses: 69.5%.

The previous generic multiple-imputation description was removed because it did not accurately represent the revised primary analysis.

A complete quantitative missingness table and analysis handling are now provided in Supplementary Table S2.

Changes made: Methods Section 2.6; Supplementary Table S2; Supplementary Figure S1; sensitivity analysis in Supplementary Table S6.

Major Comment 6 – Residual confounding and causal interpretation

Comment: The reviewer noted that several clinically important factors were unavailable and that the reported associations could be affected by residual confounding.

Response: We agree and have substantially strengthened this limitation.

The revised Discussion now explicitly identifies several clinically important variables that were unavailable or insufficiently characterized in the analytic archive, including continuous baseline spherical equivalent, axial length, magnitude of parental refractive error, detailed information on low-dose atropine, orthokeratology and other myopia-control interventions, individual-level socioeconomic indicators, and objective measurements of behavioural exposures.

These factors could be associated with both recorded behaviours and subsequent refractive progression and therefore represent potential sources of residual confounding.

We have consequently removed or softened causal language throughout the manuscript. The observed estimates are now consistently described as associations/correlates, rather than protective effects, risk effects, or evidence supporting causal behavioural interventions.

The Conclusions now explicitly state that the findings should not be used to make causal behavioural claims.

Changes made: Abstract; Results interpretation; Discussion; Limitations; Conclusions.

Minor Comments

Minor Comment 1 – Construction and interpretation of behavioural indices

Comment: The reviewer requested clearer definitions of the near-work, screen-time, device-time, outdoor, and reading-behaviour indices.

Response: We agree. We now provide the exact construction of each composite index:

• near_work_index = school classes + extra classes + self-study;

• screen_time_index = iPad/tablet use + phone use + computer use + television viewing;

• device_time_index = iPad/tablet use + phone use + computer use;

• outdoor_index = outdoor sports + outdoor leisure;

• reading_risk_index = close-reading indicator + reading-in-bed indicator.

During this audit, we also determined that the de-identified archive does not contain the original response anchors corresponding to the full 1–7 coding scale of the component variables. We have therefore avoided interpreting these scores as hours/day.

They are now described consistently as archive-specific ordinal-sum scores, and a one-unit odds ratio refers only to one score unit. This limitation is stated explicitly in the Methods and Supplementary Material.

Changes made: Methods Section 2.4; Supplementary Table S1; Supplementary Figure S2.

Minor Comment 2 – Numeric category labels

Comment: The reviewer requested that numerical categories be decoded into clinically understandable labels.

Response: We agree and have implemented this throughout the manuscript. The verified labels for sex, ethnicity, urbanicity, geographic region, socioeconomic category, baseline myopia severity, and parental myopia are now presented directly rather than as codes 1/2/3.

For example, Table 1 now reports “Male/Female,” “Urban/Rural,” “Lowland/coastal/Midland/Mountainous,” and “No parent/One parent/Both parents” instead of numerical categories.

A complete mapping is provided in Methods Section 2.5 and Supplementary Table S1.

Changes made: Methods Section 2.5; Table 1; Table 2; Supplementary Table S1.

Minor Comment 3 – School urbanicity and socioeconomic status

Comment: The reviewer questioned the inclusion and interpretation of multiple school-context variables.

Response: Thank you. During the revision we formally examined these variables and found that school urbanicity and school socioeconomic category were perfectly concordant in all 200 observations:

• 160 students were classified as both Urban and Better-off;

• 40 students were classified as both Rural and Average;

• no discordant observations were present.

The corresponding Cramér’s V is 1.000.

Because these variables contain exactly the same two-group partition in this dataset, including them simultaneously would be redundant and potentially destabilize the model.

We therefore use school urbanicity only in the revised primary model, while socioeconomic category is retained for descriptive reporting. Their exact concordance is explicitly documented in Supplementary Table S3 and Supplementary Figure S3.

Changes made: Methods Sections 2.5 and 2.7; Table 1; Supplementary Table S3 and Figure S3.

Minor Comment 4 – STROBE and TRIPOD reporting

Comment: The reviewer requested reporting according to appropriate observational and prediction-model reporting guidelines.

Response: We agree. The revised manuscript is explicitly aligned with STROBE for the observational association analysis and TRIPOD for the prediction component.

Completed STROBE and TRIPOD reporting checklists have been prepared as Supporting Information. We also clarify in the Methods that TRIPOD applies specifically to the cross-school prediction component, whereas the primary association analysis is reported according to STROBE principles.

Changes made: Introduction; Methods Sections 2.1 and 2.9; separate STROBE and TRIPOD Supporting Information checklists.

--- THE END ---

Attachment Submitted filename: Response.docx

# Peer review: Decision Letter 1

**DOI:** 10.1371/journal.pone.0358487.r003

**Authors:** Andrzej Grzybowski

1 Sep 2026

Behavioural Correlates of Higher 12-Month Myopia Progression in a High-Risk Vietnamese Schoolchild Cohort and the Limits of Cross-School Prediction

PONE-D-26-11479R1

Dear Dr. Nguyen,

We’re pleased to inform you that your manuscript has been judged scientifically suitable for publication and will be formally accepted for publication once it meets all outstanding technical requirements.

Within one week, you’ll receive an e-mail detailing the required amendments. When these have been addressed, you’ll receive a formal acceptance letter and your manuscript will be scheduled for publication.

An invoice will be generated when your article is formally accepted. Please note, if your institution has a publishing partnership with PLOS and your article meets the relevant criteria, all or part of your publication costs will be covered. Please make sure your user information is up-to-date by logging into Editorial Manager at Editorial Manager® and clicking the ‘Update My Information' link at the top of the page. For questions related to billing, please contact billing support.

If your institution or institutions have a press office, please notify them about your upcoming paper to help maximize its impact. If they’ll be preparing press materials, please inform our press team as soon as possible -- no later than 48 hours after receiving the formal acceptance. Your manuscript will remain under strict press embargo until 2 pm Eastern Time on the date of publication. For more information, please contact onepress@plos.org.

Kind regards,

Andrzej Grzybowski

Academic Editor

PLOS One

**Comments to the Author**

Reviewer #2: All comments have been addressed

**********

2. Is the manuscript technically sound, and do the data support the conclusions?

Reviewer #2: Yes

**********

3. Has the statistical analysis been performed appropriately and rigorously?

Reviewer #2: Yes

**********

4. Have the authors made all data underlying the findings in their manuscript fully available?

The PLOS Data policy

Reviewer #2: Yes

**********

5. Is the manuscript presented in an intelligible fashion and written in standard English?

Reviewer #2: Yes

**********

Reviewer #2: The authors have substantially revised the manuscript and, in my opinion, have addressed the majority of the concerns raised during the previous round of review. The revised manuscript is considerably clearer, the statistical approach is more appropriate for the available dataset, and the interpretation of the findings has been substantially moderated. I appreciate that the authors did not attempt to preserve conclusions that were no longer supported after reanalysis.

Overall, I believe the authors have responded carefully and constructively to the previous comments. The major methodological limitations of the underlying dataset remain, particularly the uncertain provenance and discretization of the myopia-progression outcome, but these limitations cannot be corrected retrospectively and are now presented with substantially greater transparency.

**********

what does this mean?). If published, this will include your full peer review and any attached files.

If you choose “no”, your identity will remain anonymous but your review may still be made public.

**Do you want your identity to be public for this peer review?** For information about this choice, including consent withdrawal, please see our Privacy Policy

Reviewer #2: No

**********

# Editor report: Acceptance letter

**DOI:** 10.1371/journal.pone.0358487.r004

**Authors:** Andrzej Grzybowski

PONE-D-26-11479R1

PLOS One

Dear Dr. Nguyen,

I'm pleased to inform you that your manuscript has been deemed suitable for publication in PLOS One. Congratulations! Your manuscript is now being handed over to our production team.

At this stage, our production department will prepare your paper for publication. This includes ensuring the following:

* All references, tables, and figures are properly cited

* All relevant supporting information is included in the manuscript submission,

* There are no issues that prevent the paper from being properly typeset

You will receive further instructions from the production team, including instructions on how to review your proof when it is ready. Please keep in mind that we are working through a large volume of accepted articles, so please give us a few days to review your paper and let you know the next and final steps.

Lastly, if your institution or institutions have a press office, please let them know about your upcoming paper now to help maximize its impact. If they'll be preparing press materials, please inform our press team within the next 48 hours. Your manuscript will remain under strict press embargo until 2 pm Eastern Time on the date of publication. For more information, please contact onepress@plos.org.

You will receive an invoice from PLOS for your publication fee after your manuscript has reached the completed accept phase. If you receive an email requesting payment before acceptance or for any other service, this may be a phishing scheme. Learn how to identify phishing emails and protect your accounts at https://explore.plos.org/phishing.

If we can help with anything else, please email us at customercare@plos.org.

Thank you for submitting your work to PLOS One and supporting open access.

Kind regards,

PLOS One Editorial Office Staff

on behalf of

Dr. Andrzej Grzybowski

Academic Editor

PLOS One
